# Neutropenia predicts better prognosis in patients with metastatic gastric cancer on a combined epirubicin, oxaliplatin and 5-fluorouracil regimen

**DOI:** 10.18632/oncotarget.5730

**Published:** 2015-10-16

**Authors:** Rujiao Liu, Mingzhu Huang, Xiaoying Zhao, Wei Peng, Si Sun, Jun Cao, Dongmei Ji, Chenchen Wang, Weijian Guo, Jin Li, Jiliang Yin, Xiaodong Zhu

**Affiliations:** ^1^ Department of Medical Oncology, Fudan University Shanghai Cancer Center, Shanghai, P.R. China; ^2^ Department of Oncology, Shanghai Medical College, Fudan University, Shanghai, P.R. China

**Keywords:** metastatic gastric cancer, EOF5 regimen, chemotherapy-induced neutropenia, overall survival, progression-free survival

## Abstract

Chemotherapy-induced neutropenia (CIN) reportedly indicated better prognosis for some cancers. We retrospectively analyzed 150 evaluable metastatic gastric cancer (MGC) patients who had received first-line EOF5 (combination regimen of epirubicin, oxaliplatin and 5-day continuous infusion of 5-fluorouracil) treatment. We divided patients into three groups according to the worst grade of CIN: absent group (grade 0), moderate group (grade 1–2) and severe group (grade 3–4). Multivariate analyses of overall survival (OS) proved moderate and severe CIN were important prognostic factors whether regarding CIN as a time-varying covariate (TVC) or not. Compared with absent CIN, hazard ratio (HR) for moderate and severe CIN were 0.31 (95% confidential interval (CI): 0.17–0.55; *P* < 0.001) and 0.36 (95% CI: 0.20–0.64; *P* = 0.001) respectively with TVC; and were 0.31 (95% CI: 0.17–0.56; *P* < 0.001) and 0.34 (95% CI: 0.19–0.61; *P* < 0.001) respectively without TVC. In progression-free survival (PFS) analyses, moderate and severe CIN showed similar results. In the landmark group (*n* = 122 patients) analyses with TVC, moderate and severe CIN remained prognostic factors for PFS, while only moderate CIN was prognostic factor for OS. CIN predicted longer OS and PFS in MGC patients treated with first-line EOF5 chemotherapy.

## INTRODUCTION

Gastric cancer is the fourth most common malignancy and the second most common cause of cancer-related death worldwide [1], and currently no standard first-line treatment regimen for gastric cancer is in place. For decades, fluoropyrimidine and platinum-based dual or triplet therapies have been the most widely used regimens for metastatic gastric cancer (MGC). The combination of epirubicin, cisplatin and 5-fluorouracil (ECF) and its modifications have shown efficacy and manageable toxicity [2], but only about 50% patients respond to it [3]. Identification of factors that predict efficacy in order to improve clinical outcome is necessary.

Reportedly, chemotherapy-induced neutropenia (CIN) was a predictor of longer survival in various types of cancer [4–9]. Possibly, CIN signified an adequate dose of anti-tumor drugs. However, patients have been receiving body surface area (BSA)-based standard dosages, which were established by small-scale dose-finding trials. Thus standard dosages may be too small for optimal efficacy in some patients, or too large to avoid unnecessary adverse effects in others [10]. And Bergh J *et al*'s study strongly supported individualized toxicity tailored chemotherapy, with a superior clinical outcome to routine chemotherapy in that study [11].

Since 2005, we performed two studies—a pilot study and an extended-sample phase II study—to evaluate the efficacy and toxicity of the EOF5 regimen (combination of epirubicin, oxaliplatin and 5-day continuous infusion of 5-fluorouracil), a modification of ECF regimen. Our results indicated that EOF5 was an effective regimen with mild toxicity, and was a suitable first-line treatment for MGC [12, 13]. To identify whether CIN was associated with better clinical outcomes, and whether it could be used to optimize treatment schedules in MGC patients who received EOF5 regimen as first-line treatment, we conducted this retrospective study.

## RESULTS

### Patients' characteristics and CIN

Among all 150 patients, 30 patients (20%) did not experience CIN (grade 0), 54 patients (36.0%) developed moderate CIN (grade 1–2), and 66 patients (44.0%) developed severe CIN (grade 3–4). Of the 120 patients who developed CIN, the highest grade of CIN occurred during the first cycle in 28 patients, the second cycle in 32 patients, the third cycle in 21 patients, the fourth cycle in 19 patients, the fifth cycle in 9 patients and the sixth cycle in 11 patients; thus 83% went through their severest CIN within 4 cycles. The median relative dose-intensity (RDI) was 0.90, indicating good compliance of EOF5 treatment. Characteristics of the study population stratified by their worst CIN grade were shown in Table 1. The groups did not significantly differ in initial characteristics. The median number of cycles of EOF5 administrated in the absent neutropenia group (4, range 1–7) was lower than that in the moderate group (6, range 2–8) or severe group (6, range 2–8), but the RDI was higher in the absent group.

**Table 1:** Patients' characteristics according to the highest grade of CIN developed during first-line chemotherapy

Features		All, *n* (%)	Absent (grade 0), *n* (%)	Moderate (grade 1–2), *n* (%)	Severe (grade 3–4), *n* (%)	*P* value
**CIN**		150 (100.0)	30 (20.0)	54 (36.0)	66 (44.0)	
**Age (years) Median age: 52 y**	**≤ median**	84 (56.0)	17 (56.7)	34 (63.0)	33 (50.0)	0.362
	**> median**	66 (44.0)	13 (43.3)	20 (37.0)	33 (50.0)	
**Sex**	**Male**	92 (61.3)	21 (70.0)	37 (68.5)	34 (51.5)	0.090
	**Female**	58 (38.7)	9 (30.0)	17 (31.5)	32 (48.5)	
**Differentiation grade**	**Low/undifferentiated**	98 (65.3)	22 (73.3)	30 (55.6)	46 (69.7)	0.309
	**Moderate/high**	15 (10.0)	1 (3.3)	8 (14.8)	6 (9.1)	
	**Unclassified**	37 (24.7)	7 (23.3)	16 (29.6)	14 (21.2)	
**Synchronous metastasis**	**Presence**	124 (82.7)	27 (90.0)	45 (83.3)	52 (78.8)	0.399
	**Absent**	26 (17.3)	3 (10.0)	9 (16.7)	14 (21.2)	
**Liver metastasis**	**No**	87 (58.0)	14 (46.7)	33 (61.1)	40 (60.6)	0.372
	**Yes**	63 (42.0)	16 (53.3)	21 (38.9)	26 (39.4)	
**Lung metastasis**	**No**	137 (91.3)	27 (90.0)	50 (92.6)	60 (90.9)	0.909
	**Yes**	13 (8.7)	3 (10.0)	4 (7.4)	6 (9.1)	
**Ascites and/or pleural effusion**	**No**	111 (74.0)	23 (76.7)	42 (77.8)	46 (69.7)	0.564
	**Yes**	39 (26.0)	7 (23.3)	12 (22.2)	20 (30.3)	
**Baseline hemoglobin Median Hb: 120 g/L**	**≤ Median**	77 (51.3)	11 (36.7)	28 (51.9)	38 (57.6)	0.164
	**> Median**	73 (48.7)	19 (63.3)	26 (48.1)	28 (42.4)	
**Baseline platelet count Median count: 241 × 10^9^/L**	**≤ Median**	75 (50.0)	11 (36.7)	25 (46.3)	39 (59.1)	0.100
	**> Median**	75 (50.0)	19 (63.3)	29 (53.7)	27 (40.9)	
**No. of cycles of EOF5 administrated: median (range)**		6 (1–8)	4 (1–7)	6 (2–8)	6 (2–8)	
**Relative dose-intensity: median**		0.90	0.93	0.90	0.89	

### Kaplan-Meier survival analyses according to the worst grade of CIN

The median overall survival (OS) of absent, moderate and severe group was 6.83 months (95% confidential interval (CI) 5.31–8.35), 19.07 months (95%CI 13.93–24.21) and 11.33 months (95%CI 8.20–16.94), respectively (Figure 1A). Furthermore, the median progression-free survival (PFS) of absent, moderate, and severe group was 3.10 months (95%CI 2.18–4.02), 7.90 months (95%CI 4.94–11.31) and 6.07 months ((95%CI 5.51–6.63), respectively (Figure 1B). These results supported experience of CIN was associated with better prognosis.

**Figure 1A:**
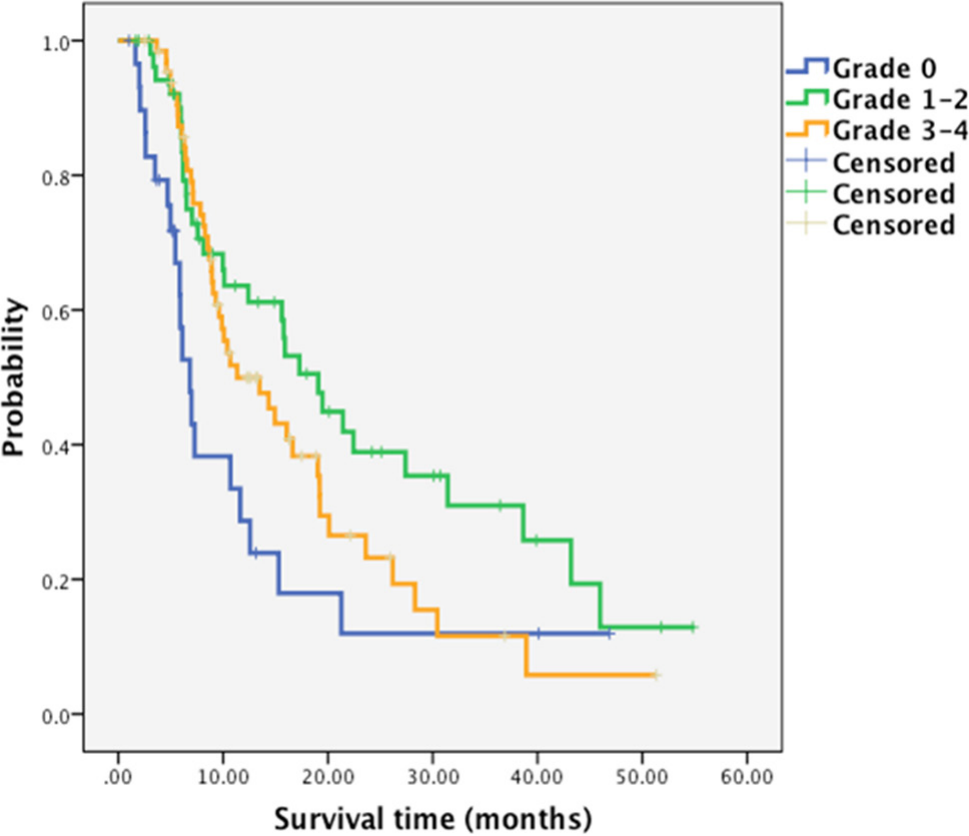
Kaplan-Meier survival curves by patients' worst grade of chemotherapy-induced neutropenia Median overall survival, absent group: 6.83 months (95%CI 5.31–8.35); moderate group: 19.07 months (95%CI 13.93–24.21); and severe group: 11.33 months ((95%CI 8.20–16.94), respectively.

**Figure 1B:**
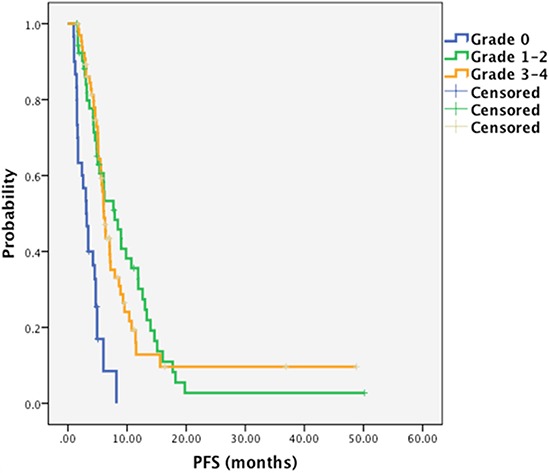
Kaplan-Meier survival curves by patients' worst grade of chemotherapy-induced neutropenia Median PFS, absent group: 3.10 months (95%CI 2.18–4.02); moderate group: 7.90 months (95%CI 4.94–11.31); and severe group: 6.07 months ((95%CI 5.51–6.63), respectively.

### Association of CIN and OS adjusted with other variables

Univariate Cox analyses suggested that low/undifferentiated tumors, liver metastasis, lung metastasis, ascites and/or pleural effusion, baseline hemoglobin less than median (120 g/L), and absent CIN were associated or tended to be associated with higher risk of death (*P* < 0.1; Table 2). The hazard ratio (HR) for moderate, severe CIN relative to absent CIN was 0.43 (95% CI: 0.24–0.75; *P* = 0.003), 0.59 (95% CI: 0.35–1.01; *P* = 0.053), respectively.

**Table 2:** Univariate analyses (UA) and multivariate analyses (MA) of overall survival with or without time-varying covariate (TVC)

Baseline and clinical features		UA without TVC			MA without TVC			MA with TVC		
		HR	95% CI	*P*	HR	95% CI	*P*	HR	95% CI	*P*
**CIN**	**Absent**	1.00			1.00			1.00		
	**Moderate**	0.43	0.24–0.75	**0.003**	0.31	0.17–0.56	**< 0.001**	0.31	0.17–0.55	**< 0.001**
	**Severe**	0.59	0.35–1.01	0.053	0.34	0.19–0.61	**< 0.001**	0.36	0.20–0.64	**0.001**
**Differentiation grade**	**Low/undifferentiated**	1.00			1.00			1.00		
	**Moderate/high**	0.51	0.25–1.04	0.065	0.72	0.34–1.50	0.373	0.72	0.34–1.50	0.377
	**Unclassified**	0.92	0.57–1.47	0.718	0.94	0.58–1.55	0.816	0.95	0.58–1.55	0.832
**Liver metastasis**		1.48	0.98–2.23	0.060	2.55	1.60–4.06	**< 0.001**	2.54	1.59–4.04	**< 0.001**
**Lung metastasis**		2.00	1.03–3.89	0.040	2.05	1.04–4.05	0.039	2.03	1.03–4.01	**0.042**
**Ascites and/or pleural effusion**		2.55	1.63–3.98	**< 0.001**	4.09	2.42–6.90	**< 0.001**	4.06	2.40–6.85	**< 0.001**
**Baseline hemoglobin > median (120 g/L)**		0.68	0.45–1.03	0.070	0.66	0.43–1.01	0.057	0.66	0.43–1.02	0.061

After a stepwise backward elimination of those 6 potential prognostic factors in a multivariate model, 4 covariates remained significantly: absent CIN, liver metastasis, lung metastasis, and ascites and/or pleural effusion. Both moderate and severe CIN favored significantly longer OS than did absent CIN, with HR of 0.31 (95% CI: 0.17–0.56, *P* < 0.001), and 0.34 (95% CI: 0.19–0.61; *P* < 0.001), respectively. When CIN was treated as a time-varying covariate (TVC), absent CIN, liver metastasis, lung metastasis, and ascites and/or pleural effusion remained significant prognostic factors. The HR for moderate, severe CIN compared with absent CIN was 0.31 (95% CI: 0.17–0.55; *P* < 0.001), 0.36 (95% CI: 0.20–0.64; *P* = 0.001), respectively.

### Association of CIN and PFS adjusted with other variables

Univariate Cox regression analyses identified 6 covariates as potentially favorable prognostic factors for PFS (*P* < 0.1; Table 3): moderate or severe CIN, moderate/highly differentiated tumors, heterochronous metastasis, no ascites and/or pleural effusion, baseline hemoglobin > median (120 g/L), and baseline platelet count > median (241 × 10^9^/L; Table 3).

**Table 3:** Univariate analyses (UA) and multivariate analyses (MA) of progression-free survival with or without time-varying covariate (TVC)

Baseline and clinical features		UA without TVC			MA without TVC			MA with TVC		
		HR	95% CI	*P*	HR	95% CI	*P*	HR	95% CI	*P*
**CIN**	**Absent**	1.00			1.00			1.00		
	**Moderate**	0.22	0.13–0.38	**< 0.001**	0.21	0.12–0.37	**< 0.001**	0.23	0.13–0.40	**< 0.001**
	**Severe**	0.25	0.15–0.42	**< 0.001**	0.23	0.14–0.39	**< 0.001**	0.29	0.17–0.50	**< 0.001**
**Differentiation grade**	**Low/undifferentiated**	1.00			1.00			1.00		
	**Moderate/high**	0.34	0.16–0.74	**0.007**	0.51	0.23–1.15	0.105	0.52	0.23–1.16	0.110
	**Unclassified**	0.88	0.58–1.35	0.570	0.85	0.54–1.32	0.465	0.86	0.55–1.34	0.500
**Heterochronous (relapse from radical surgery)**		0.41	0.24–0.72	**0.002**	0.50	0.29–0.88	**0.016**	0.49	0.28–0.85	**0.012**
**Ascites and/or pleural effusion**		1.91	1.28–2.84	**0.002**	2.08	1.38–3.14	**< 0.001**	2.00	1.33–3.00	**0.001**
**Baseline hemoglobin > median (120 g/L)**		0.70	0.48–1.02	0.060	0.75	0.51–1.09	0.129	0.76	0.52–1.11	0.157
**Baseline platelet count > median (241 × 10^9^/L)**		1.52	1.05–2.21	**0.028**	1.15	0.77–1.73	0.487	1.10	0.72–1.68	0.648

In multivariate Cox regression analysis, CIN remained significant, as did heterochronous metastasis, and ascites and/or pleural effusion. Compared with absent CIN, the HR for moderate CIN was 0.21 (95% CI: 0.12–0.37; *P* < 0.001), and for severe CIN was 0.23 (95% CI: 0.14–0.39; *P* < 0.001), indicating longer PFS for patients with moderate CIN or severe CIN. In multivariate regression analysis that treated CIN as a TVC, moderate/severe CIN, heterochronous metastasis, and no ascites or pleural effusion remained associated with improved PFS. Compared with absent CIN, the HR for moderate CIN and severe CIN was 0.23 (95% CI: 0.13–0.40; *P* < 0.001), and 0.29 (95% CI: 0.17–0.50; *P* < 0.001), respectively.

### Survival analyses in the landmark cohort

At last, we conducted landmark analyses, limiting to 122 patients who received at least four cycles of chemotherapy and were alive 90 days after initiating EOF treatment (Table 4). When treating CIN during that period as a baseline feature adjusted with other variables, moderate and severe CIN favored significantly longer PFS in comparison to absent CIN, with an HR of 0.35 (95% CI: 0.20–0.64; *P* = 0.001), 0.43 (95% CI: 0.23–0.81; *P* = 0.009), respectively. But moderate/severe CIN showed no association with OS.

**Table 4:** Multivariate analyses of prognostic factors of OS and PFS (landmark group, *n* = 122)

Baseline and clinical features		OS (without TVC)			OS (with TVC)			PFS (without TVC)			PFS (with TVC)		
		HR	95% CI	*P*	HR	95% CI	*P*	HR	95% CI	*P*	HR	95% CI	*P*
**CIN**	**Absent**	1.00			1.00			1.00			1.00		
	**Moderate**	0.59	0.31–1.13	0.109	0.44	0.21–0.95	**0.035**	0.35	0.20–0.64	**0.001**	0.24	0.12–0.48	**< 0.001**
	**Severe**	1.01	0.51–1.98	0.982	0.66	0.32–1.38	0.267	0.43	0.23–0.81	**0.009**	0.31	0.16–0.62	**0.001**
**Differentiation grade**	**Low/undifferentiated**	1.00			1.00			1.00			1.00		
	**Moderate/high**	0.70	0.33–1.48	0.352	0.75	0.36–1.58	0.445	0.66	0.29–1.49	0.314	0.63	0.28–1.42	0.264
	**Unclassified**	0.92	0.52–1.63	0.778	0.92	0.52–1.62	0.771	0.86	0.52–1.45	0.577	0.83	0.50–1.38	0.465
**Heterochronous (relapse from radical surgery)**								0.42	0.22–0.78	**0.006**	0.44	0.24–0.83	**0.011**
**Liver metastasis**		2.05	1.23–3.41	**0.006**	2.01	1.20–3.38	**0.008**						
**Lung metastasis**		2.33	1.05–5.17	**0.038**	2.30	1.03–5.12	**0.041**						
**Ascites and/or pleural effusion**		3.49	1.95–6.24	**< 0.001**	3.58	1.97–6.52	**< 0.001**	2.04	1.27–3.27	**0.003**	1.92	1.20–3.09	**0.007**
**Baseline hemoglobin > median (120.5 g/L)**		0.71	0.43–1.16	0.175	0.76	0.47–1.24	0.268	0.71	0.46–1.10	0.129	0.75	0.49–1.15	0.183
**Baseline platelet count > median (241 × 10^9^/L)**								0.94	0.57–1.53	0.792	1.00	0.62–1.62	0.996

When considering CIN as a TVC adjusted with other variables in landmark analyses, both moderate CIN (HR: 0.24; 95% CI: 0.12–0.48; *P* < 0.001) and severe CIN (HR: 0.31; 95% CI: 0.16–0.62; *P* = 0.001) were identified as a favorable prognostic factors for PFS. In OS analyses, moderate CIN favored significantly longer OS, with an HR of 0.44 (95% CI: 0.21–0.95; *P* = 0.035), while severe CIN showed no significant advantage (HR: 0.66; 95% CI: 0.32–1.38; *P* = 0.267).

### Clinical outcomes of patients without CIN and with grade 1 CIN during first two cycles

Totally 49 patients did not develop CIN during the first two cycles of treatment, and among these patients, 30 (61.2%) did not developed CIN during the following cycles of treatment, and only 6 patients (12.2%) developed grade 3 CIN (no grade 4 CIN). Those who developed grade 1 CIN within the first 2 cycles were likely to have longer survival than those with no CIN (HR: 0.56; 95% CI: 0.31–1.03; *P* = 0.060), and had a significantly longer PFS (HR: 0.50; 95% CI: 0.30–0.84; *P* = 0.009).

## DISCUSSION

In this study, we retrospectively investigated the relationship between CIN and clinical outcome in MGC patients who received EOF5 treatment as first-line therapy, showing that experience of CIN predicted longer survival and PFS compared with no CIN. However, the prognostic role of neutropenia has seldom been reported in a triple-combined regimen for MGC patients.

Issues about the statistical methods were concerned. Actually, patients who survived longer received more cycles of EOF chemotherapy, and thus had greater chances of developing CIN, hence, considering CIN as a baseline feature could produce a false-positive association between CIN and overall survival, TVC and landmark analyses were approaches applied for fear of this problem [6, 7, 14, 15]. In the present study, the two methods were employed. We conducted landmark analyses, limiting to 122 patients who received at least four cycles of chemotherapy and were alive 90 days after initiation of EOF treatment, with the highest grade of CIN during that period as a baseline feature. However, some patients continued to receive EOF chemotherapy after four cycles unless disease progression or intolerable toxicity happened, which led to late-onset CIN. In the present study, 20 patients had their worst grade of CIN in the fifth or sixth cycle of chemotherapy. Consequently, such landmark analyses ignored the information of those whose CIN occurred after the landmark. Furthermore, CIN was considered as a TVC in landmark cohort analyses to adjust the bias of CIN that occurred after the landmark; those with moderate CIN had consistently longer OS and PFS in the landmark cohort TVC analyses.

Our results were consistent with the aforementioned studies [5–9] in which we found the favorable prognostic role of CIN. The underlying mechanism of CIN as an optimistic prognostic factor could be explained, by that CIN reflected the response of hematopoietic stem cells to chemotherapy drugs, which depended on drug concentration partly. Hence, CIN severity could be a measure of the plasma drug concentration. In other words, lack of bone marrow suppression might indicate that a chemotherapy dosage is too low to produce a strong biological effect.

In addition, severe CIN displayed a consistently higher HR than moderate CIN in both OS and PFS analyses, suggesting patients with severe CIN got less survival benefits from chemotherapy than those with moderate CIN. Differed from our study, in some previous studies, patients with severe CIN (grade 3–4) showed a lower HR than patients with moderate CIN (grade 1–2), reaping greater survival benefits [6, 15]. Similar with our study, Han Y *et al*. reported that patients with severe CIN had a higher HR of death (HR: 0.64; 95% CI: 0.42–0.98; *P* = 0.038) among breast cancer patients who received an ECF triplet regimen [5].

Severe CIN might be associated with poor reserved bone marrow function and deficient tolerance, especially in a relatively aggressive triplet regimen. Despite of the active G-CSF support in the present study (G-CSF was permitted to use when grade 2 neutropenia was identified), 11 patients went through grade 4 neutropenia, suggesting deficient chemotherapy tolerance. This led to higher incidences of chemotherapy delay and dose reduction, with a relative lower RDI (0.88), which might result in a higher hazard ratio of severe neutropenia.

The question of whether CIN, as a favorable prognostic factor, could optimize individualized dosages warranted investigation. The current widely used dose determination by BSA ignored inter-individual differences and thus could not always determine the optimal dosage. Reasons for this individual variation were unidentified, although genetic polymorphisms that affect drug metabolism or elimination might contribute [16–18]. Dose adjustments based on pharmacokinetic monitoring have reportedly led to a significantly improved clinical outcomes compared with BSA-guided dosages [11, 19–21]. Pharmacokinetic data showed that BSA-tailored dosage for fluoropyrimidine might result in over- or under-dosing [21–23]. Oxaliplatin and epibubicin might have similar situations [24–26]. However, pharmacokinetic monitoring was not practical in daily clinical work.

In our study, among patients without CIN during the first two cycles, 61.2% did not developed CIN during the following cycles of treatment, representing an unfavorable prognostic factor. Furthermore, patients who developed grade 1 CIN in the first two cycles had significantly longer PFS, and tended to have enhanced OS than those with no CIN. These findings implied that the initial BSA-based doses could be fine-tuned for patients without CIN in the first two cycles, aiming at experience of CIN in the latter four cycles of EOF chemotherapy. A dose increase could thus be implemented early in treatment for patients with no CIN after the first and second cycles. Due to the relative small size and retrospective nature of the present study, the efficacy of such a strategy could be evaluated in prospective trials.

## MATERIALS AND METHODS

### Patients

This was a retrospective study. All 150 patients in this study were evaluable, they were treated with EOF5 regimen in a phase II trial conducted in our center (http://ClinicalTrials.gov ID: NCT00767377). Principal inclusion criteria and treatment administration for patients were described previously [13]. Written informed consent was obtained from all patients. The study was approved by the Institutional Review Board of the Fudan University Shanghai Cancer Center.

### Evaluation of survival and CIN

During the phase II study, complete blood cell counts (CBC) were detected twice a week. Prophylactic application of granulocyte-colony-stimulating factor (G-CSF) was not allowed unless patient developed grade ≥ 2 neutropenia. We identified the severest grade of CIN based on the lowest recorded neutrophil count. CIN was graded according to the National Cancer Institute Common Toxicity Criteria version 3.0. Overall survival (OS) was defined as the internal between the date of enrollment and the date of death or last follow-up. Progression-free survival (PFS) was calculated from the date of enrollment to the date of disease progression or death before progression.

### Statistical methods

The primary endpoint of this study was the association of CIN with OS for patients treated with EOF5 regimen. The second endpoint was the association between CIN and PFS. Patients were classified into different groups according to their severest grade of CIN. Differences in clinical characteristics between groups were compared using χ^2^ or Fisher's exact probability tests. Dose intensity was calculated as the total dose received per unit of body surface area per unit of time (mg/m^2^/day), and RDI was the ratio between actual dose intensity and scheduled dose intensity. Survival curves were done by the Kaplan–Meier method and analyzed by the log-rank test. Several baseline characteristics (liver metastasis, lung metastasis, baseline hemoglobin, etc.) and CIN were analyzed as prognostic factor candidates; a reduced model was applied using stepwise backward elimination until only significant (*P* < 0.05) variables remained. However, as CIN varies over time, CIN was analyzed both as an initiated factor and as a time-varying covariate (TVC). TVC was identified as the worst grade of CIN that occurred between the initiation of EOF chemotherapy and time *T* > 0. We also used landmark analyses limited to 122 patients who received at least four cycles of chemotherapy and were alive 90 days after initiation of EOF treatment. All tests were two-sided. *P* < 0.05 was considered significant. Analyses were performed using the SPSS 10.0 software (Chicago, IL, USA).

## CONCLUSIONS

For MGC patients who received EOF5 regimen as first line treatment, those experienced CIN with EOF5 treatment had better OS and PFS. Fine-tuning of the initial BSA-based dose for patients without CIN in the first two cycles merits further prospective trial.

## Abbreviations

CIN: Chemotherapy-induced neutropenia
MGC: Metastatic gastric cancer
EOF5: Combination regimen of epirubicin, oxaliplatin, and 5-day continuous infusion of 5-fluorouracil
OS: Overall survival
PFS: Progression-free survival
HR: Hazard ratio
ECF: Combination regimen of epirubicin, cisplatin, and 21-day continuous infusion of 5-fluorouracil
BSA: Body surface area
RECIST: Response evaluation criteria in solid tumors
TVC: Time-varying covariate
CI: Confidential interval
RDI: relative dose-intensity
